# A Real-Time Zanthoxylum Target Detection Method for an Intelligent Picking Robot under a Complex Background, Based on an Improved YOLOv5s Architecture

**DOI:** 10.3390/s22020682

**Published:** 2022-01-17

**Authors:** Zhibo Xu, Xiaopeng Huang, Yuan Huang, Haobo Sun, Fangxin Wan

**Affiliations:** College of Mechanical and Electrical Engineering, Gansu Agricultural University, Lanzhou 730070, China; xuzb@st.gsau.edu.cn (Z.X.); huangxp@gsau.edu.cn (X.H.); huangy@st.gsau.edu.cn (Y.H.); sunhb@st.gsau.edu.cn (H.S.)

**Keywords:** Zanthoxylum, artificial intelligence, YOLOv5, target detection, picking robot

## Abstract

The target recognition algorithm is one of the core technologies of Zanthoxylum pepper-picking robots. However, most existing detection algorithms cannot effectively detect Zanthoxylum fruit covered by branches, leaves and other fruits in natural scenes. To improve the work efficiency and adaptability of the Zanthoxylum-picking robot in natural environments, and to recognize and detect fruits in complex environments under different lighting conditions, this paper presents a Zanthoxylum-picking-robot target detection method based on improved YOLOv5s. Firstly, an improved CBF module based on the CBH module in the backbone is raised to improve the detection accuracy. Secondly, the Specter module based on CBF is presented to replace the bottleneck CSP module, which improves the speed of detection with a lightweight structure. Finally, the Zanthoxylum fruit algorithm is checked by the improved YOLOv5 framework, and the differences in detection between YOLOv3, YOLOv4 and YOLOv5 are analyzed and evaluated. Through these improvements, the recall rate, recognition accuracy and mAP of the YOLOv5s are 4.19%, 28.7% and 14.8% higher than those of the original YOLOv5s, YOLOv3 and YOLOv4 models, respectively. Furthermore, the model is transferred to the computing platform of the robot with the cutting-edge NVIDIA Jetson TX2 device. Several experiments are implemented on the TX2, yielding an average time of inference of 0.072, with an average GPU load in 30 s of 20.11%. This method can provide technical support for pepper-picking robots to detect multiple pepper fruits in real time.

## 1. Introduction

The Zanthoxylum pepper is one of the most widely planted cash crops in China. Traditionally, it is mainly picked by hand with high cost, high labor intensity, low efficiency, low security and strong seasonal timing. In addition, Zanthoxylum is mostly planted on ridges and convex ridges with low fertility, making picking more difficult and time-consuming. Thus, low picking efficiency has seriously restricted the economic benefit and development of the Zanthoxylum industry. To realize efficient automatic picking of Zanthoxylum fruit, reducing the burden of forest garden pickers and ensuring the timely picking of fruit, it is significant to carry out in-depth research on the key technologies of Zanthoxylum-picking robots. Therefore, rapid real-time detection under natural conditions without the influence of a complex environment has very important application value and practical significance to improve the operation efficiency of picking robots.

With the continuous development of artificial intelligence, artificial neural networks have been widely used in many fields, and deep learning target detection methods based on target detection have been gradually applied, surpassing traditional image-processing methods [1,2,3]. Artificial intelligence continues to progress and be widely used in different fields. For example, in the economic field, a deep neural network model can be combined with sample data and feature engineering to estimate stock price changes [4]. By combining artificial neural networks and grey correlation analysis, the purchasing intention of consumers in the exchange can be analyzed and predicted [5]. In the industrial field, Danyang Zhang et al. [6] proposed a multiobject detection method based on deep convolution combined with relevant ideas of neural networks, which can realize nondestructive detection of rail surfaces and fastener defects. Haifeng Wang et al. [7] proposed a traffic sign YOLO (TS-YOLO) model based on a convolutional neural network to improve the detection and recognition accuracy of traffic signs under conditions of extremely limited vision. Gang Tang et al. [8] proposed an excellent ship detection method named “N-YOLO”, which was based on YOLO, including a noise level classifier (NLC), SAR target potential area extraction module (STPAE) and detection module based on YOLOv5. Benwu Wang et al. [9] proposed a deep network detection method based on X-ray images to detect abnormalities in the molding process of industrial inserts. Liu et al. [10] used frequency-domain-focusing technology of synthetic aperture radar (SAR) to aggregate scattered GPR signals and obtain test images. The noise in the original signal is removed by a designed low-pass filter, and the target contour is extracted by edge detection using background information. In the field of agriculture, a new deep learning structure, VddNet (Vine Disease Detection Network), was proposed to detect grape diseases [11]. Real-time identification of early fusarium wilt in potato production systems was achieved using machine vision combined with deep learning technology [12]. Ji et al. [13] used an SVM classifier to classify and recognize apple fruits, and the recognition rates of bagged fruits reached 89%. X. Wei et al. [14] extracted a new color feature from the OHTA color space and used the improved Otsu algorithm to automatically calculate the segmentation threshold of fruit images, with a recognition accuracy of more than 95%. Yao Jia et al. [15] proposed a defect detection model based on YOLOv5, which could quickly and accurately detect defects in kiwifruit with mAP@0.5 reaching 94.7%. Bin Yan et al. [16] proposed a lightweight apple target detection method for picking robots based on improved YOLOv5s, with a recognition recall rate of 91.48%, recognition accuracy of 83.83%, mAP of 86.75% and F1 of 87.49%. Yangyu et al. [17] proposed a new strawberry-picking robot and fruit pose estimator named Rotating YOLO (R-YOLO), which significantly improved the positioning accuracy of picking points, with an average recognition rate of 94.43% and recall rate of 93.46%. All these studies provided strong evidence and broad prospects for the application of artificial intelligence in modern agriculture; however, the universality and robustness, which were provided by samples and human subjectivity, have not been processed.

Zanthoxylum fruit target detection is similar to the majority of target detection programs in many aspects, such as UAVS automatic navigation, fire detection and face recognition. Therefore, traditional detection models, such as R-CNN [18,19,20], Faster R-CNN [21], YOLO [22,23,24,25] and SSD [26], have been applied to the detection of Zanthoxylum. Among these models, R-CNN, SSP-NET and Faster R-CNN have two detection stages, with high accuracy but much slower computing speed than YOLO and SSD models with primary structures. YOLO (You Look Only Once) includes YOLO, YOLOv3 [27,28,29,30,31], YOLOv4 [32] and YOLOv5 [33]. Other methods are favored by researchers because they could directly train the target position in single-stage operation. Detection based on artificial neural networks and computer vision technology can provide faster, more real-time and more efficient detection for agricultural robots in target growth monitoring, moisture monitoring and target location extraction. However, the above models cannot quickly and efficiently provide accurate positioning for picking robots in complex orchard environments. Thus, a highly robust target detection system based on computer vision and a fully autonomous automatic detection model of UAV [34,35,36] systems is of urgent need. On the other hand, though the identification efficiency of most Zanthoxylum target recognition research models based on deep learning is high, the timeliness and accuracy of the models is insufficient in complex orchard environments with different fruit sizes and serious branch clustering. Therefore, it is of great importance to develop a method that can simultaneously recognize multiple clusters of fruits, meet the application requirements in a complex forest environment, and detect Zanthoxylum fruits in real time.

Therefore, this work put forward a lightweight Zanthoxylum-pepper-targeted real-time recognition algorithm, which is based on the improved YOLOv5, and thus provide reliable technical support for the picking robot to realize the real-time and efficient detection of Zanthoxylum peppers in a complex forest environment. The main contributions of this work are summarized as follows:

(1) As Zanthoxylum is a multicluster fruit with strong randomness of growth direction, we adopted the deep learning method in computer vision, which is not often tried in multicluster fruit. A set of complete detection algorithms was established, which provided a method for picking robots to identify and detect fruit in forest gardens.
(2) Considering the multicluster nature of Zanthoxylum fruit, a detection module with the addition of the FReLU activation function was adopted to effectively improve the efficiency and accuracy of fruit recognition. By changing the CSP module in the backbone, a lightweight Specter module was proposed to accelerate the convergence speed of the training network and reduce the impact on the scale loss.
(3) In consistent environmental tests, the real-time detection of several classical target detection networks of Zanthoxylum fruit on the running platform of the robot, an NVIDIA Jetson TX2, was compared and analyzed. Based on YOLOv5, the feature extraction and multiscale detection of the network were enhanced and the training parameters were reduced. Good results were achieved in the Zanthoxylum fruit dataset.

## 2. Materials and Methods

### 2.1. Zanthoxylum Fruit Image Collection

#### 2.1.1. Material and Image Data Collection

**Figure 1:**
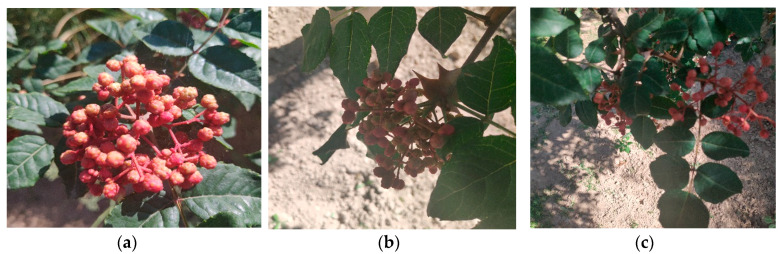

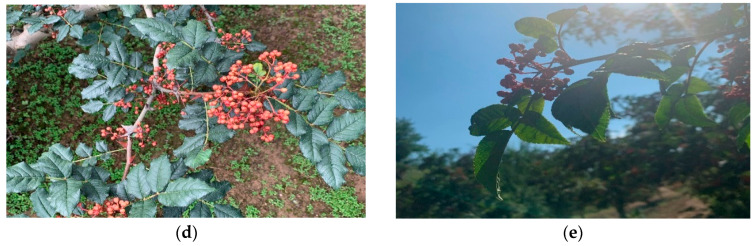
Images of Zanthoxylum under different conditions. (**a**) Single cluster of Zanthoxylum with smooth light and no shade; (**b**) Single cluster of Zanthoxylum with backlight; (**c**) Zanthoxylum with shade of leaves; (**d**) Clusters of Zanthoxylum with overlapping smooth fruits; (**e**) Clusters of Zanthoxylum with backlight.

This study takes the fruit of the Zanthoxylum tree in a modern Zanthoxylum garden as the research object. As shown in Figure 1, the original images were collected from the Industrial Park of Maiji District, Tianshui City, Gansu Province; and the Zanthoxylum Park of Jishi Mountain, Dongxiang County, Gansu Province, respectively. Pepper trees in the garden row were spaced approximately 3 meters apart, plant spacing was approximately 1.8 meters, and the tree height was approximately 2 meters, which was suitable for the pepper-picking robot to work in the garden. The Zanthoxylum varieties were Dahongpao and Mianjiao. All the JPEG images are collected by Nikon 40D camera, and all the image resolutions are 6000 × 4000 pixels. A total of 4000 prickly pepper fruit images were collected.

#### 2.1.2. Image Preprocessing

Object detection based on deep learning was trained on a large amount of image data. The dataset was enhanced in order to obtain enriched image training set, better extract image features and avoid overfitting. Firstly, 2800 images were randomly selected from 4000 images as the training set, 800 images were set as the test set and 400 images were chosen to be the verification set. The detailed distribution of the testing set is shown in Table 1. Secondly, the image resolution was reduced to 3024 × 3024 pixels to reduce the running time of subsequent tests, and LabeLImg was used to label the images manually. The smallest enclosing rectangle of each Zanthoxylum fruit string was labeled to ensure that there was only one Zanthoxylum fruit in each labeling frame. Thus, the background was kept as minimal as possible. Furthermore, all the generated XML files were saved and converted to TXT files.

**Figure 2:**
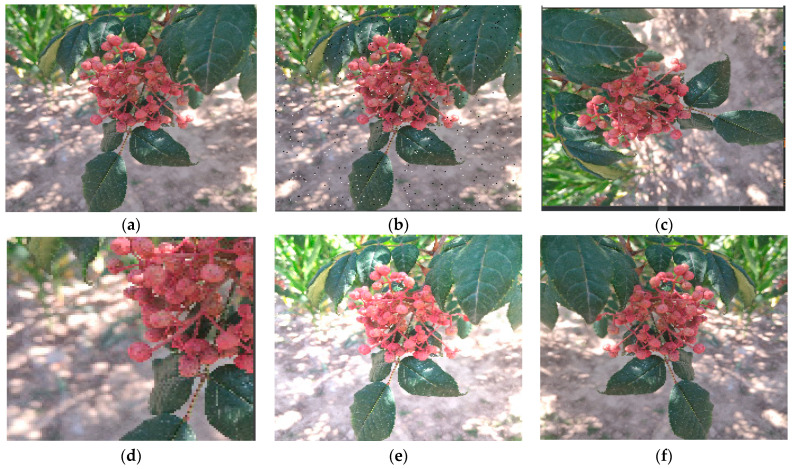
Image enhancement results. (**a**) Ripe Zanthoxylum string; (**b**) Increased noise result; (**c**) Rotation result; (**d**) Random clipping result; (**e**) Increased brightness result; (**f**) Mirror flip result.

Due to the complex lighting conditions during image acquisition, the original image was processed based on the image processing operations of OpencV and related libraries, in order to improve the generalization ability of the training model (Figure 2). The process was carried out in five ways, including image brightness enhancement, image rotation, image mirror flip, image random clipping and image noise increase. Rotated images, random clipping, increased noise and flipped images can improve the detection performance and robustness of the network. Meanwhile, increased brightness can eliminate the impacts of the brightness deviation on network performance caused by the environmental lighting changes and sensor differences. After data augmentation, the image among the 20,000 images was randomly selected according to 7:2:1 for deep network training and parameter verification, without overlap, to avoid overfitting of the training model.

### 2.2. Improvement of YOLOv5s Network Architecture

#### 2.2.1. YOLOv5

At present, the main target recognition algorithms are R-CNN and YOLO. R-CNN is widely used with high accuracy but cannot meet the requirements of real-time rapid detection for picking robots. Thus, YOLO is the better choice, as it can quickly regress the image information in a simple channel and at the same time, classify and observe the target detection information. In this work, YOLOv5, as the latest algorithm in the YOLO series with fast training speed, high detection accuracy and small model weight file, was employed. It contains four architectures, YOLOv5s, YOLOv5m, YOLOv5l and YOLOv5x, and the architecture size differs with the difference in the convolution kernel size and the feature extraction times. 

The accuracy and real-time performance of the Zanthoxylum fruit detection model are crucial to the real-time operational efficiency of the Zanthoxylum picking robot were ensured.

**Figure 3:**
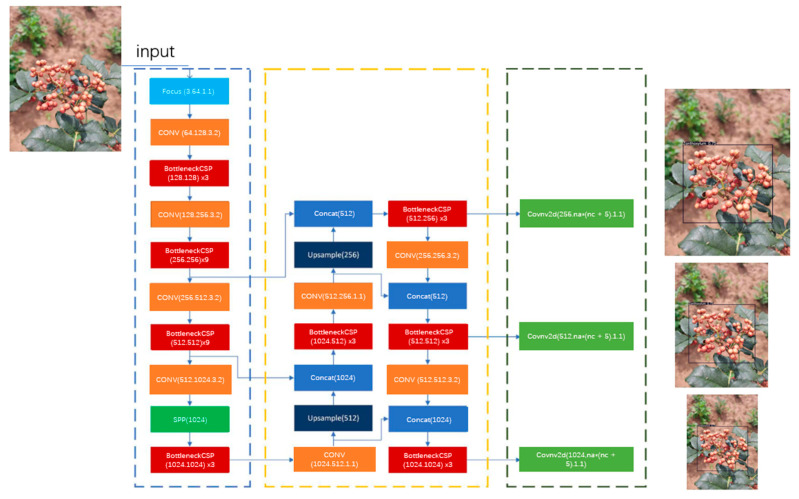
YOLOv5s framework.

The YOLOv5s framework consists of a backbone, neck and head. The backbone aggregates the input image information by different types of image granularity to form the convolutional neural network of image features. The neck transmits the output image of the backbone layer to the prediction layer in a pyramid mixed structure. The head generates prediction boxes and categories based on image features transmitted by the neck, as shown in Figure 3.

#### 2.2.2. Improvement of Backbone Network

The recognition algorithm of the Zanthoxylum-picking robot must not only accurately identify Zanthoxylum fruit in the complex environment of the Zanthoxylum forest park, but also be built in the hardware of the robot with a lightweight model by optimizing and improving the backbone based on YOLOv5s. Because the edge contour of Zanthoxylum fruit is irregular, the FReLU activation function was adopted [37] to improve the accuracy. Under the premise of ensuring detection accuracy, the parameter volume and number of network weights were reduced to realize the lightweight improvement of the fruit target detection network of the Zanthoxylum-picking robot.

**Figure 4:**
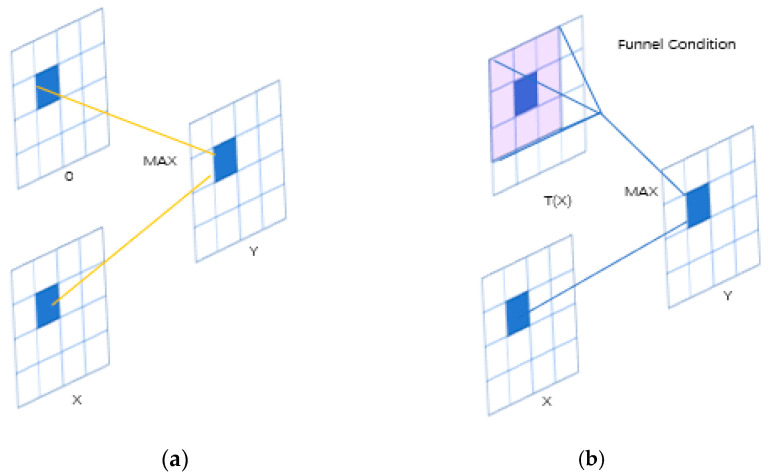
FReLU for visual recognition. (**a**) ReLU: MAX(X,0); (**b**) FReLU: MAX(X,T(X)).

The FreLU activation function is based on the ReLU activation function, and adopts the simple nonlinear function *Max* (), which can be extended by adding a visual funnel condition *T*(*x*) to connect each pixel to the 2D environment, as shown in Figure 4.

(1) $$F\left(x_{C},I,j\right)=MAX\left(x_{C},I,j,T\left(x_{C},I,j\right)\right)$$

(2) $$T\left(x_{C},i,j\right)=x_{c,i,j}^{\omega }P_{c}^{\omega }$$

In Formulas (1) and (2), *Xc*, *I*, *j* represents the input pixel of the nonlinear activation function on channel C. At the 2D space position (*I*, *j*), the function *T*() represents the funnel condition, *X_c_*, *I*, *j,ω* represents *kh* × *kW* centered on *Xc*, and $p_{c}^{\omega }$ represents the coefficients shared by this window in the same channel.

**Figure 5:**
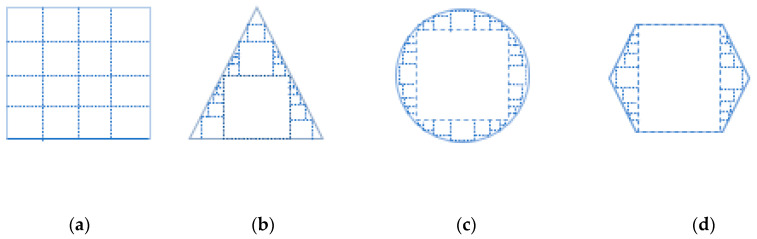
Activation fields of different shapes. (**a**) Normal activation field; (**b**) Oblique shape; (**c**) Arc shape; (**d**) Multi-oblique shape.

As shown in Figure 5, squares of different sizes represent different activation fields for each pixel in the activation layer at the top. For example, in Figure 5a, each pixel is a square activation field with the same size; in Figure 5b, there are square activation fields of different sizes; and in Figure 5c,d, curved and oblique shapes are more common object outlines in fruits. Therefore, the original Hardwish activation function was replaced by the FReLU activation function, and irregular and detailed pixel data could be better captured in the complex Zanthoxylum fruit detection training by using funnel-modeling ability during training.

**Figure 6:**
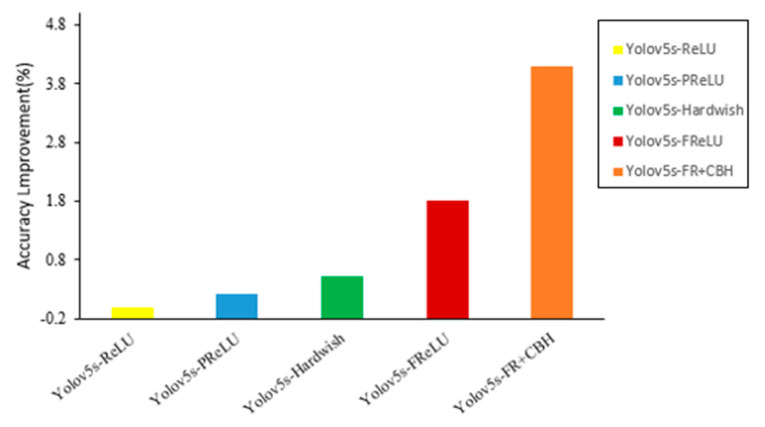
Comparison of different versions of YOLOv5s.

Usually, immediately after capturing the spatial dependency in a convolution layer linearly, an activation layer acts as a scalar nonlinear transformation. Many insightful activations have been proposed, such as ReLU, PReLU, Hardwish and FReLU, but improving their performance on visual tasks is challenging. Therefore, currently, the most widely used activation is still ReLU. We set the ReLU network as the baseline and show the relative improvement in accuracy on the basic tasks in computer vision: object detection (mAP). As shown in Figure 6, we trained YOLOv5s over the Zanthoxylum dataset to evaluate the generalization performance of the model on this dataset. FReLU is more effective and transfers better on the tasks.

**Figure 7:**
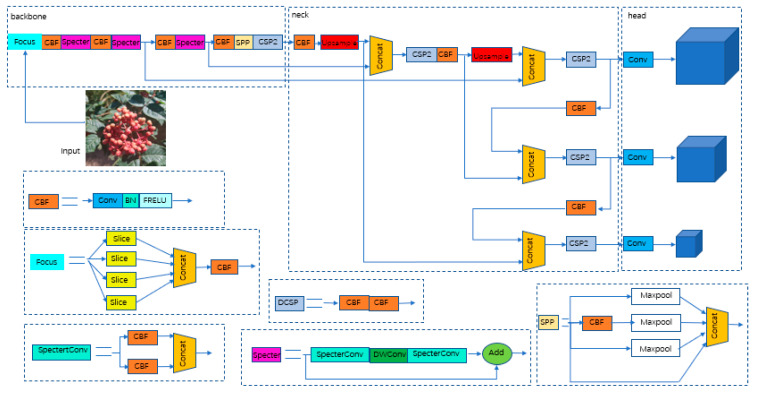
Improved YOLOv5 network.

**Figure 8:**
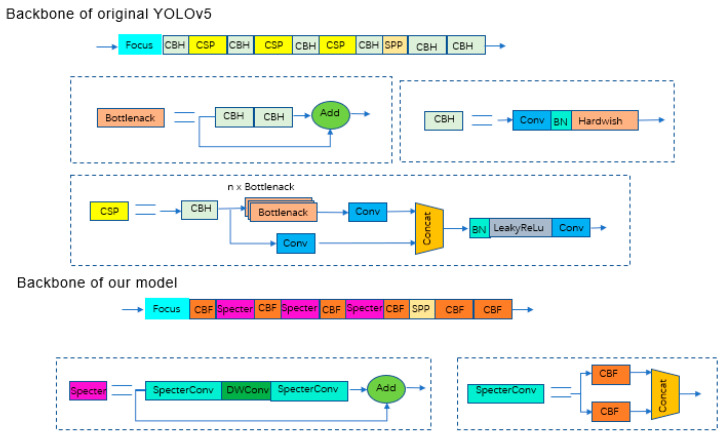
Backbone before and after improvement. The CBH module is composed of the Conv (convolution), BN (batch normalization) and Hardwish activation functions.

The original YOLOv5s network utilizes cross-stage partial (CSP) to increase the network depth and thus improve the feature and detection capability of the network. However, during the detection task of Zanthoxylum fruit in natural Zanthoxylum gardens, it was found that some lightweight computing models can also achieve satisfactory test results and reduce memory operation to facilitate installation in mobile robots. As shown in Figure 7 and Figure 8, to improve network detection speed and reduce the model size, a Specter bottleneck based on the Ghost bottleneck was used instead of a CSP bottleneck in the original network. Conv (convolution), BN (batch normalization) and FReLU compose the CBF module, which is the basic part of SpectertConv. Specifically, the input of SpectertConv enters two CBF modules, and the outputs of those modules concatenate in the channel dimension to be the output of SpectertConv. The core idea of Specter is to generate a large number of feature graphs with rich Zanthoxylum information, by using low-cost convolution operations. First, a few conventional convolution operations were performed on the feature graph to generate the basic features. Then, more features were generated using the deep convolutional network, which were finally combined with the basic features to generate the final output features.

**Figure 9:**
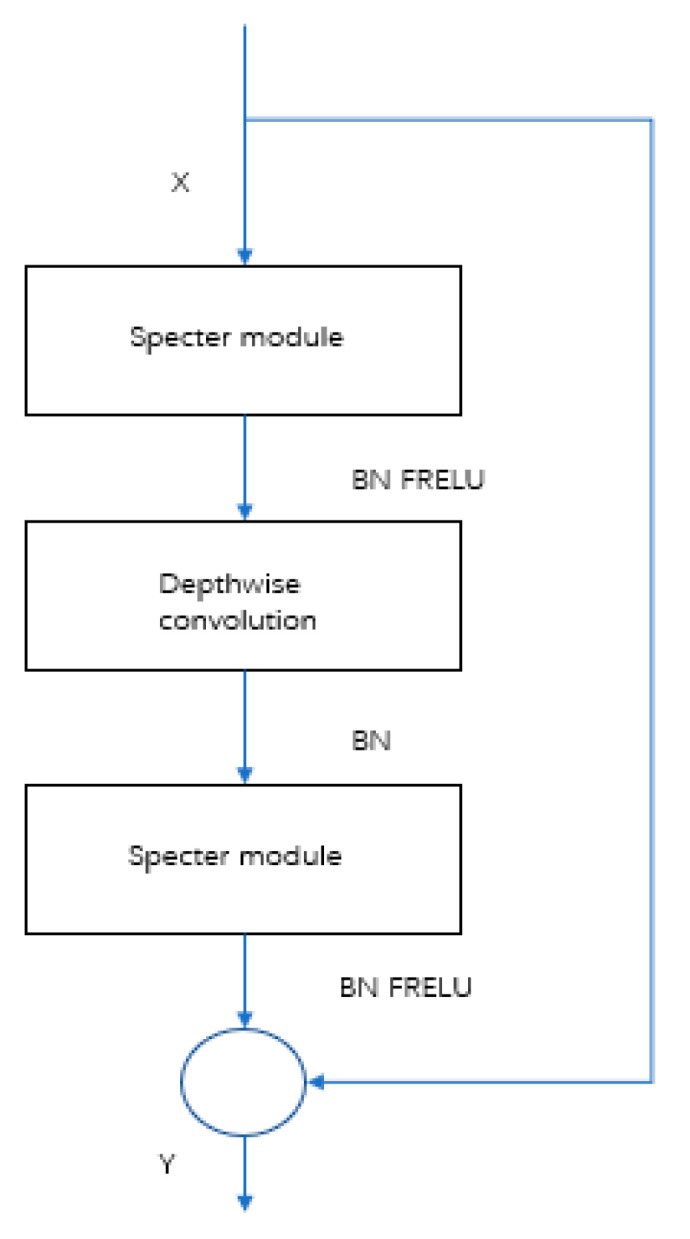
Specter bottleneck.

The structure of the Specter bottleneck is shown in Figure 9. It consists of two Specter modules. In this network model, the number of channels was first increased by the Specter module, then features were integrated by deep convolution. Finally, the number of channels was adjusted by the Specter module, which was the same as the number of channels in another process, and added to obtain the output feature information. The Specter module could change the number of input channels by changing the number of convolution kernels. To effectively reduce parameter redundancy and increase the geometric characteristic information of prickly pepper fruit, a convolution layer was added to the two Specter modules. BN was added after the convolutional layer of each module, and the FReLU [37] activation function was added after the convolutional layer of the two Specter modules to improve the expressive ability of the neural network.

### 2.3. Network Training

#### 2.3.1. Platforms

In this experiment, the PyTorch deep learning framework was built on the hardware platform of an AMD Ryzen7 5800H CPU (16 GB of memory) and an NVIDIA GeForce RTX3060 Laptop GPU (6 GB of video memory) under the Windows 10 operating system. CUDA Cudnn, OpenCV and related libraries were called to implement the target detection model of a Zanthoxylum fruit-picking robot, trained and tested.

In the real-time detection process, the trained model was implemented on the platform of the robot with the cutting-edge NVIDIA Jetson TX2 device. The TX2 equipped with an NVIDIA Pascal^TM^ GPU with 256 NVIDIA CUDA cores provides superior speed and efficiency. Moreover, the module size of the TX2 is only 50 mm × 87 mm, which meets the space–size requirements of the robot control platform.

In this study, the batch size was set as 24, and the weights of the model were regularized and updated by the BN layer. The momentum was set as 0.937, and the weight decay rate (decay) was set as 0.0005. The initial vector and IOU thresholds were set as 0.01, and the enhancement coefficients of hue (H), saturation (S) and brightness (V) were set as 0.015, 0.7 and 0.4, respectively. The number of the training epochs was set as 900, and every message was recorded for each training. After the training, the weight files of the recognition model were saved, and the performance of the model was evaluated by the test set. The final output of the network was the predicted position box of the identified Zanthoxylum fruit.

#### 2.3.2. Training Results

**Figure 10:**
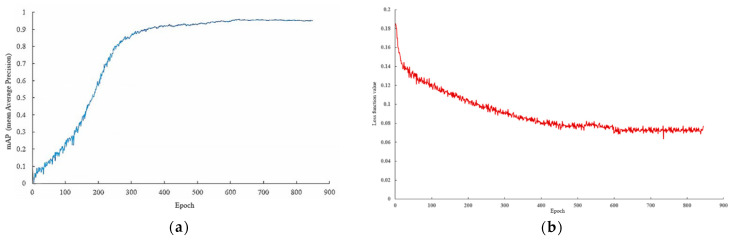
(**a**) mAP curve; (**b**) Loss curve.

The mAP (mean average precision) of the training set is displayed in Figure 10a; Figure 10b shows the loss curve of the training process, indicating that the loss value decreased rapidly in the first 150 epochs and tended to be stable after 600 epochs. The training was good, and no fitting occurred. Therefore, the output model of training 900 epochs was determined as the fruit target detection model of the Zanthoxylum-picking robot in this study.

## 3. Experimentation and Results

### 3.1. Model Evaluation Index

In this study, precision P (precision)—namely, accuracy, recall and mAP—were used to evaluate the performance of the detection model.

### 3.2. Experimental Results

**Figure 11:**
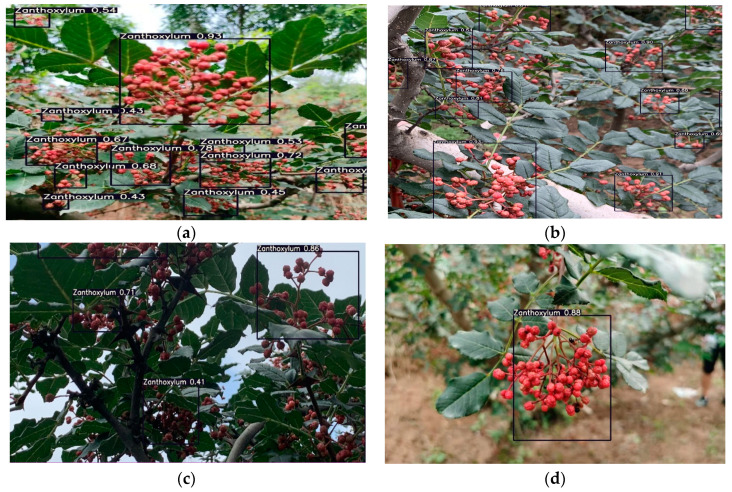
Recognition results of Zanthoxylum by the improved YOLOv5s network in the morning. (**a**) Multiple clusters of blocked Zanthoxylum fruit. (**b**) Multiple clusters of unblocked Zanthoxylum fruit. (**c**) Single cluster of Zanthoxylum fruit under backlight. (**d**) Single cluster of Zanthoxylum fruit under sunlight.

**Figure 12:**
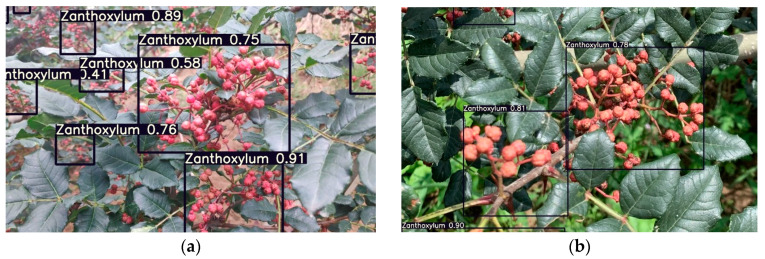

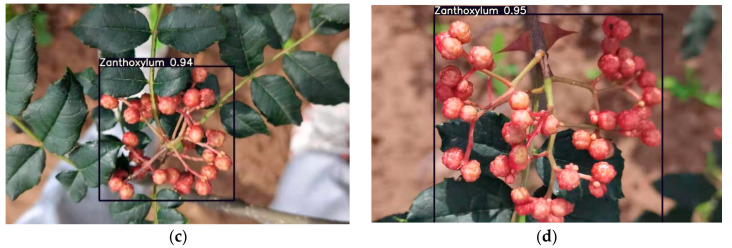
Recognition results of Zanthoxylum by the improved YOLOv5s network in the afternoon. (**a**) Multiple clusters of blocked Zanthoxylum fruit. (**b**) Multiple clusters of unblocked Zanthoxylum fruit. (**c**) Single cluster of Zanthoxylum fruit under backlight. (**d**) Single cluster of Zanthoxylum fruit under sunlight.

**Figure 13:**
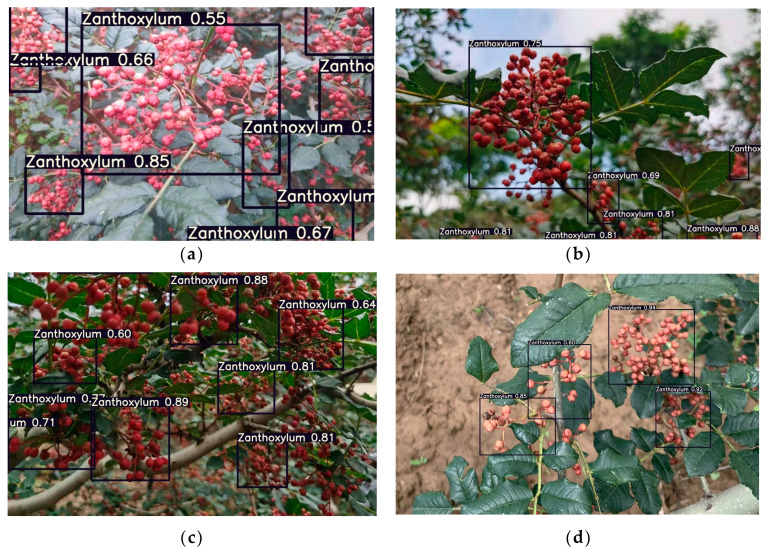
Recognition results of Zanthoxylum by the improved YOLOv5s network under different illumination environment on TX2 platform. (**a**) Multiple clusters of blocked Zanthoxylum fruit. (**b**) Single cluster of Zanthoxylum fruit under backlight. (**c**) Multiple clusters of blocked Zanthoxylum fruit. (**d**) Single cluster of Zanthoxylum fruit under backlight.

To verify the performance of the optimized network model for Zanthoxylum fruit detection, this study designed a real-time identification model of a Zanthoxylum-picking robot, which was based on the improved YOLOv5s. The optimized network model was applied in 4000 images, and the detection results in multiple clusters of blocked Zanthoxylum fruits, multiple clusters of unblocked Zanthoxylum fruits and a single cluster of Zanthoxylum fruits under different lighting conditions were carefully analyzed. The mAP of this model is 94.5%. As shown in Figure 11, Figure 12 and Figure 13, it can be seen that in the early morning environment, the natural light is weak, and a small number of multicluster pepper fruits cannot be fully identified; in the afternoon environment, the natural light is strong, and most pepper fruits can be well identified and detected. Overall, the identification results of the improved YOLOv5s network proposed in the study were accurate.

### 3.3. Comparison of the Recognition Results of Different Target Detection Algorithms

To further analyze the recognition performance of the proposed algorithm for Zanthoxylum fruit, the improved YOLOv5s network was compared with the original YOLOv5s, YOLOv3-TINY and YOLOv4-TINY networks on 2000 verification set images. The mAP value and average recognition speed of the model were used as evaluation indexes. The identification results, size and number of parameters of each network model are shown in Table 2.

**Figure 14:**
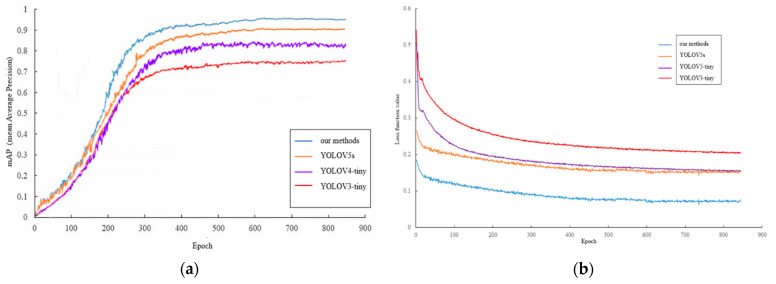
Comparison of different detection models. (**a**) mAP curve; (**b**) Loss curve.

According to Table 2 and Figure 14, the mAP value of the improved YOLOv5 recognition model proposed in this paper is the highest, which is 4.19% higher than that of the original YOLOv5 network, 28.7% higher than that of YOLOv3-TINY and 14.8% higher than that of YOLOv4-TINY, respectively. The results showed that the algorithm is the best among the four methods. The average detection speed of the improved YOLOv5s model is 0.012 s/image, which is 1.25 times, 1.42 times and 2.5 times those of the original YOLOv5, YOLOv4-TINY and YOLOv3-TINY networks, respectively. All these showed that the model can meet the requirements of the picking robot for real-time identification of Zanthoxylum. On the other hand, Table 2 and Figure 12 showed that the size of the improved YOLOv5s recognition model proposed in this paper is only 14.0 MB, accounting for 97.2%, 60.6% and 41.5% of the original YOLOv5s, YOLOv4-TINY and YOLOv3-TINY networks, respectively. The results demonstrated that the network could not only ensure the recognition accuracy but also effectively realize the lightweight characteristics of the network. In general, the model proposed in this study is the lightest among the five network models with the highest mAP value. The recognition speed of this model is better than that of YOLOv3-TINY, the original YOLOv5s and YOLOv4-TINY, which could meet the requirement of real-time Zanthoxylum fruit recognition. Further analysis can be obtained in Table 2. On the TX2 platform, the inference speed of our model was the fastest, the speed is 33.23 FPS, and the average load of the GPU was the lowest in these models. The irregular and detailed pixel data of pepper fruit are better captured by FReLU activation function, and a large number of feature maps with rich pepper information are generated by low-cost convolution operation. Firstly, some conventional convolution operations are performed on the feature map to generate the basic features. Then, more features are generated by using the deep convolution network, and are finally combined with the basic features to generate the final output features, which effectively improve the detection speed and performance of our network.

## 4. Conclusions

In this paper, a method that could effectively detect and recognize Zanthoxylum fruit in natural scenes is proposed. Based on the YOLOv5s algorithm and FReLU activation function, the method greatly improved the integrity of pepper fruit information and the quality of the training set. A Specter module was proposed to replace the bottleneck CSP module to improve the detection speed with a lightweight structure. In addition, several classical target detection networks were compared and analyzed for real-time detection of Zanthoxylum pepper fruit. Based on these improvements, the feature extraction and multiscale detection of the network were significantly enhanced, and the training parameters were reduced. Good results were achieved in the Zanthoxylum fruit dataset. In future work, we will focus on the main branch of Zanthoxylum fruit and integrate the picking point location algorithm with the main branch detection algorithm, in order to achieve real-time localization and detection of Zanthoxylum fruit-picking points.

## Figures and Tables

**Table 1:** Sample analysis details of text set images.

Conditions	Morning		Afternoon	
	Frontlighting	Backlighting	Frontlighting	Backlighting
Number of images	195	186	225	194
Graspable Zanthoxylum	588	564	563	285
Ungraspable Zanthoxylum	547	634	535	329

**Table 2:** Comparison of different detection models in the Zanthoxylum pepper dataset.

Object Detection Networks	mAP (%)	Average Detection Speed (s/pic)	Average Detection Speed of TX2 (s/pic)	Average GPU Load on TX2(%)	Average Detection FPS of TX2	Model Size (MB)
YOLOv3-TINY	73.4	0.030	0.114	38.72	35.13	33.7
YOLOv4-TINY	82.3	0.017	0.153	27.98	22.45	23.1
YOLOv5s	90.7	0.015	0.097	24.25	28.62	14.4
Our network	94.5	0.012	0.072	20.11	33.23	14.0

## Data Availability

The raw data required to reproduce these findings cannot be shared at this time as the data also form part of an ongoing study.
